# Piezoelectric A^15^B^16^C^17^ Compounds and Their Nanocomposites for Energy Harvesting and Sensors: A Review

**DOI:** 10.3390/ma14226973

**Published:** 2021-11-18

**Authors:** Piotr Szperlich

**Affiliations:** Institute of Physics-Centre for Science and Education, Silesian University of Technology, Krasinskiego 8 Street, 40-019 Katowice, Poland; piotr.szperlich@polsl.pl

**Keywords:** A^15^B^16^C^17^ compunds, nanocomposites, piezoelectrics, energy harvesting, sensors

## Abstract

Interest in pyroelectrics and piezoelectrics has increased worldwide on account of their unique properties. Applications based on these phenomena include piezo- and pyroelectric nanogenerators, piezoelectric sensors, and piezocatalysis. One of the most interesting materials used in this growing field are A^15^B^16^C^17^ nanowires, an example of which is SbSI. The latter has an electromechanical coupling coefficient of 0.8, a piezoelectric module of 2000 pC/N, and a pyroelectric coefficient of 12 × 10^−3^ C/m^2^K. In this review, we examine the production and properties of these nanowires and their composites, such as PAN/SbSI and PVDF/SbSI. The generated electrical response from 11 different structures under various excitations, such as an impact or a pressure shock, are presented. It is shown, for example, that the PVDF/SbSI and PAN/SbSI composites have well-arranged nanowires, the orientation of which greatly affects the value of its output power. The power density for all the nanogenerators based upon A^15^B^16^C^17^ nanowires (and their composites) are recalculated by use of the same key equation. This enables an accurate comparison of the efficiency of all the configurations. The piezo- and photocatalytic properties of SbSI nanowires are also presented; their excellent ability is shown by the high reaction kinetic rate constant (7.6 min^−1^).

## 1. Introduction

**Figure 1:**
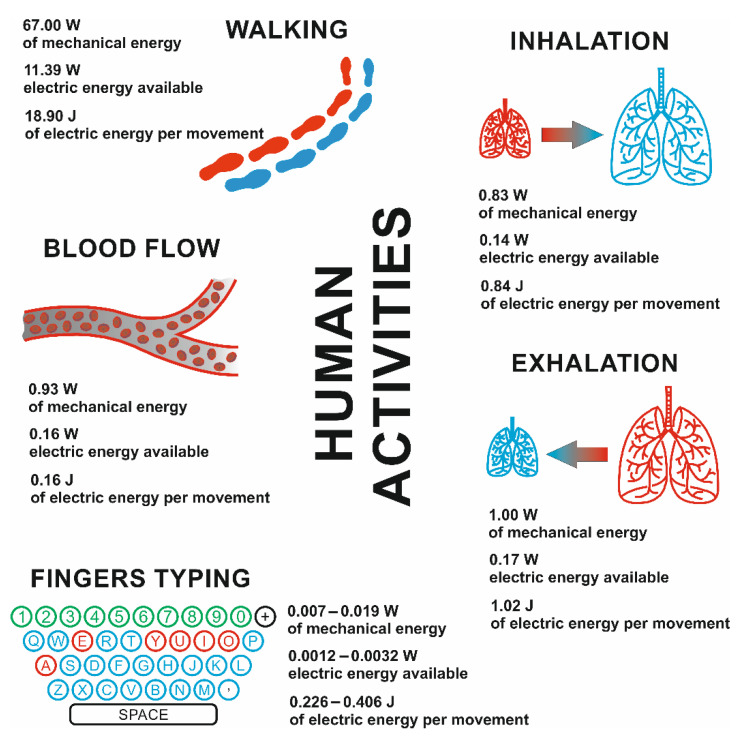
Power values for certain body motions.

The continuous development of the economy relating to, inter alia, urbanization and the growth of the global population has led to an increase in demand for energy. Due to the environmental pollution caused by existing energy sources (i.e., coal, oil, gas, etc.), renewable energy sources have become increasingly popular. Not only has this enabled enhanced production of green energy but it has also allowed us to become independent from a need to import fossil fuels and to provide energy to isolated areas without a developed energy grid. Among the many forms of useful energy that we can acquire from the natural environment, several kinds are most prominent, for example solar, thermal, and mechanical energy. One of the widely available and underrated energy sources is the motions of the human body. Our daily activities (e.g., fingers typing and walking) and the activities necessary for life (e.g., inhalation, exhalation, and blood flow) provide energy that can be converted into electrical energy. Examples of power (energy per unit time) values related to the human body are given in Figure 1 [1].

The recent high expectations for the harvesting of energy are often connected to nanodevices. This has arisen because of the continuous development of nanotechnology, and thus the increased production of novel nanomaterials that are useful to this branch of technology. Many physical phenomena are able to acquire energy from the surrounding environment, e.g., via the piezoelectric, ferroelectric-photovoltaic, pyroelectric, and triboelectric effects. In the case of nanomaterials, their physical properties may differ from bulk materials and, in conjunction with a large surface-to-volume ratio, they can be particularly valuable materials in certain configurations.

Due to the above stated phenomena and the advancement of nanomaterials, it is feasible for fabricated nanogenerators to convert various forms of energy into electric energy. It is apparent that these forms of devices are a fairly new technology. The earliest nanogenerator was constructed by Wang in 2006 and involved the use of an array of zinc oxide (ZnO) nanowires based on the piezoelectric effect [2]. Six years later, the pioneering papers on pyroelectric [3] and triboelectric [4] nanogenerators were published. Moreover, many research advances have been achieved for piezoelectric nanogenerators (PENGs) based on various nanomaterials, e.g., ZnO nanostructures [5], ZnO nanowire arrays [6,7], CdS nanowires [8,9], PZT nanofibers [10], PZT thin films [11], porous PVDF [12], lithium-doped zinc oxide nanowires-polymer composite [13], CdSe [14], and sodium-potassium niobate nanorods [15]. The accelerating development and production of nanogenerators is evidenced by the increasing number of publications on the subject; following the first publications in 2006, 200 papers appeared in 2015, then almost 500 publications in 2018, and over 1000 works were seen in 2020.

This paper provides a review on the various types of nanogenerators that use A^15^B^16^C^17^ nanowires, and their composites, based on piezoelectric and pyroelectric effects. The registered values of the voltages, currents, and power for each individual device are compared herein. The general direction for future research that applies A^15^B^16^C^17^ compounds to the fields of renewable energy sources and sensors is also proposed.

## 2. Materials and Methods

One of the most significant materials, which have a wide range of interesting properties, are A^15^B^16^C^17^ compounds. They consist of elements from three different groups of the periodic table (for example, 15 is Sb or Bi, 16 is S or Se, and 17 is Cl or I). The best-known compounds of this type are antimony sulphoiodide (SbSI) and antimony selenoiodide (SbSeI). The dominant appearance of SbSI may be because, in some publications, the whole group is called SbSI-type compounds. These materials are characterized by their covalent bonds, they can exist in both amorphous and crystalline form, and they are semiconductors with a bandgap, *E_g_*, between 1 eV and 3 eV. The latter depends on their molar composition, e.g., *E_g_* = 1.829 (27) eV for SbSI [16] and *E_g_* = 1.63 eV for SbSeI [17], respectively.

The procedure for the synthesis of SbSI, and the study of its properties were first described by Henry and Garot in 1834 [18]. The initial period of intensive research on these compounds arose in the 1950s and 1960s. The crystalline structure of A^15^B^16^C^17^ compounds, and their properties, were determined by E. Dönges in 1950 [19,20]. He discovered that these crystalline structures were comprised of double chains [(SbSI)_∞_]_2_ consisting of two chains linked by a two-fold screw axis. These two chains are connected by strong covalent Sb-S bonds, while weak van der Waals bonds (<3.8 eV) hold the double chains together. The growth rate of SbSI crystals along the *c*-axis is found to be two orders of magnitude greater than growth in the perpendicular directions; consequently, the obtained crystals are shaped similar to long needles. The photoelectric phenomenon in A^15^B^16^C^17^ compounds was discovered in 1960 [21] then, three years later, the first paper on their ferroelectric properties was published [22]. In the early 1980s, reports appeared on the photovoltaic effect in SbSI [23,24] and SbSI_0.3_Br_0.7_ [24] crystals. While in recent years, the ferro-photovoltaic effect [25,26,27,28,29] was discovered in SbSI, i.e., a photovoltage or photocurrent response that acts along the polarization direction of the SbSI crystal.

The Curie temperatures are 293 K [30] and 223 K [31] for SbSI and SbSeI, respectively. It has been determined that these temperatures can be shifted to higher values by replacing iodine with another element. For example, the Curie temperature shifts to 340 K [32] for the case of SbSI_0.8_Cl_0.2_, in which 20% of iodine atoms are replaced with chlorine atoms. However, most interest in A^15^B^16^C^17^ compounds arose because of their piezoelectric properties, which was reported by Berlincourt in 1964 [33]. He presented a value for the electromechanical coupling coefficient of the SbSI single crystal along the *c*axis (*k*_33_ = 0.8) and the maximum value of the piezoelectric module (*d*_33_ = 2 × 10^−9^ C/N), which positioned the crystal at the forefront of known piezoelectric materials. Table 1 provides a comparison of the functional properties of a few selected piezoelectric materials, including SbSI single crystal and its ceramic equivalent.

Papers on the synthesis methods for SbSI crystals can be divided into three groups: (i) growth from the vapor phase [19,43,44,45,46,47,48,49,50,51], (ii) growth from the molten mass (Bridgman [21,34,35,52,53,54,55,56,57,58,59,60], zone melting [61,62], including under increased pressure [63,64]), and (iii) crystallization from solutions [55,57,65], which includes the hydrothermal method [66,67].

Growth from the vapor phase and the Bridgman method is used for the synthesis of SbSI monocrystals with mirror-like surfaces. In the first case, the process is carried out in a closed ampule. In order to obtain the largest possible crystals, the seeds are selected in the crystallization zone by temporarily increasing the temperature in this zone. Single crystals with cross-sections of several millimetres (e.g., [48,49,51]) are produced in this way. The Bridgman method was used, among others, by Mori and Tamura [34,52]. They obtained crystals in the form of needles with dimensions 2 × 1 × 10 mm^3^ or plates with a size of 5 × 10 × 0.5 mm^3^. The largest crystals with a cross-section of 5 × 5 mm^2^ were reported in [57], however, the presented photos show the growth of many single crystals rather than a single crystal. Polycrystalline samples consisting of many crystals in the form of needles are obtained using the zone melting method, e.g., [61,62]. The growth of SbSJ from aqueous solutions is kept at a high pressure and results in crystals with cross-sections of a fraction of a millimetre, e.g., [31,66]. The main difficulty in this method is finding a solvent that allows the synthesis of larger crystals of SbSI. Much larger samples (with cross-sections greater than 1 cm) were produced in the form of hot-pressed SbSI ceramics. In this case, their physical properties were not satisfying [68].

In addition, several years ago, sonochemical synthesis technology was developed for A^15^B^16^C^17^ nanowires [16,17,69,70,71,72,73,74,75,76,77,78,79,80,81,82,83,84]. In sonochemistry, powerful acoustic waves with frequencies greater than 20 kHz are used. When an acoustic wave propagates though a liquid, the medium alternates between compressions and rarefactions. In the case of a high-intensity ultrasound, during a period of negative pressure, micro caverns can arise in the medium because the intermolecular forces are too weak to maintain the cohesion. If the liquid is volatile, or contains volatile substances, vapor can insert into the cavities and create gas-filled microbubbles that are compressed during the positive pressure period. The phenomenon of the formation, growth, and collapse of the bubbles is called cavitation. Following “hot-spot” theory, extremely high temperatures and pressures can arise inside the bubbles during their collapse. The values of these parameters were estimated at 5200 ± 650 K and 1700 atm, respectively [85]. Sonochemistry, which can be performed in liquids at room temperature and ambient pressure, can stimulate chemical reactions that require high temperatures and pressures. This method is a fast, efficient, convenient, and environmentally friendly route for the fabrication of SbSI nanocrystals in a single step. In addition, compared with the other production methods of SbSI, it is free of any explosion hazard [86].

### 2.1. A^15^B^16^C^17^ Nanowires Synthesis

**Figure 2:**
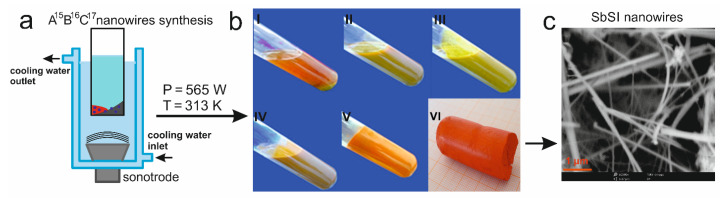
Schematic diagrams showing the synthesis technique for A^15^B^16^C^17^ nanowires. (**a**) Elements from groups 15, 16, 17 are flooded with liquid and placed into a sonochemical reactor. (**b**) Changes in color and consistency are observed during an exemplary synthesis reaction of SbSI nanowires (over the time period: I—0 min, II—20 s, III—6 min, IV—26 min, V—120 min); image (VI) is a photograph of the obtained SbSI gel. (**c**) An SEM image of the SbSI nanowires.

During the typical sonochemical synthesis of A^15^B^16^C^17^ compounds, a stoichiometric mixture of high purity elements (e.g., A = Sb or Bi; B = S or Se; C = I or Cl) [16,17,69,70,71,72,73,74,75,76,77,78,79,80,81,82] or compounds (e.g., $A_{2}^{15}B_{3}^{16}\;$ = Sb_2_S_3_, $A^{15}C_{3}^{16}$ = SbI_3_) [83] were immersed in various liquids: ethanol [16,17,69,70,71,72,73,74,75,76,83,84], methanol [77,78], isopropanol [79], or water [80,81,82]. To prevent an outflow of volatile reaction products, the substrates (within the appropriate solvent) were placed in a closed polypropylene cylinder (Figure 2a). The prepared solution was then put into the sonochemical reactor. Reactors of different powers (and power densities) and frequencies can be used, for example: Table 1 provides a comparison of the functional properties of a few selected piezoelectric materials, including SbSI single crystal and its ceramic equivalent.

During the synthesis, changes are clearly seen in both the colour and consistency of the sol (Figure 2b). The colour change is caused by the formation of compounds with different specific energy gaps; SbI_3_ appears in the first stages of the synthesis, and then SbSI is created. Since SbI_3_ has a greater optical bandgap than SbSI, the sol turns from an initial green colour to orange. The change in consistency is associated with a transformation of the sol (which contains molecules, clusters, or individual nanocrystallites) into a gel (composed of nanowires, as seen in Figure 2c). The time duration of the synthesis is dependent on the type of solvent used; the shortest times were recorded for processes performed in alcohol (e.g., 120 min for ethanol) [16,17], while longest times relate to synthesis in water (550 min) [80], both under a temperature of 298 K. This can be attributed to the large difference in iodine solubility in various liquids, e.g., 21 g/100 g and 0.0334 g/100 g in ethanol and water (at the above-mentioned temperature), respectively. As a result, the much lower solubility of iodine in water means a much slower synthesis of SbI_3_, which is a necessary intermediate step in the creation of SbSI nanowires. The obtained material was rinsed several times with ethanol and then centrifuged to remove any unreacted reagents. Technological details on the synthesis of A^15^B^16^C^17^ compounds, and their properties, can be found in refs [16,17,69,70,71,72,73,74,75,76,77,78,79,80,81,82,83,84]. Sonochemically produced A^15^B^16^C^17^ nanowires can be used to fabricate various compounds, and act as an important component in different types of nanogenerators and sensors.

### 2.2. PAN/SbSI Composite

The PAN/SbSI nanocomposite was created by the electrospinning method. The spinning solutions were prepared using the following reagents: polyacrylonitrile (PAN, Mw = 150,000 g/mole, Sigma-Aldrich, Poznan, Poland), N,N–dimethylformamide (DMF), and ethanol (EtOH, purity 99.8%, Avantor Performance Materials Poland, Gliwice, Poland). The previously obtained sonochemically-produced SbSI nanowires were used as a reinforcing phase, so that 0.7 g of SbSI nanowires were added to 8.95 mL of DMF.

**Figure 3:**
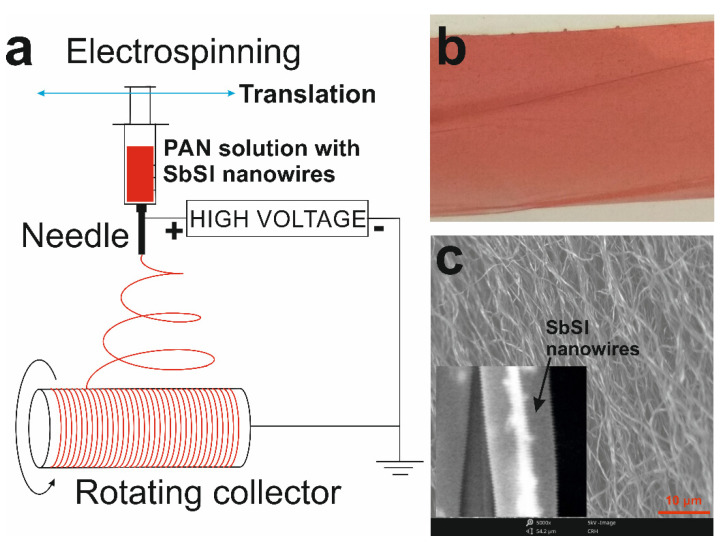
(**a**) Scheme depicting the electrospinning set-up for fabrication of PAN/SbSI fibrous mats. (**b**) An exemplary photograph and (**c**) a SEM image of PAN/SbSI composite mat. The inset of (**c**) shows a single nanofiber with of SbSI nanowires.

The prepared solutions were sonicated for 1 h, which destroys the agglomerates of the nanowires and ensures a homogeneous distribution within the produced composites. After this time, PAN (0.75 g) was added to the solution and was mixed using a magnetic stirrer for 24 h. The composites were fabricated using the FLOW Nanotechnology Solutions Electrospinner 2.2.0-500 device, which is equipped with a drum collector (Figure 3a). The following parameter values were used in the electrospinning process: the distance between the collector and the nozzle is set at 0.125 m, the speed of solution flow is 3.0 mL/h, the potential difference between the electrodes is 20 kV, and the drum collector rotation speed is 800 RPMs (for both polymers). An exemplary photograph and a SEM image of the obtained composites are presented in Figure 3b,c (PAN/SbSI). An analysis of the morphology of the obtained mats, which consists of nanofibers, indicates that the diameter of the nanofibers is constant, and the SbSI nanowires are evenly dispersed, do not agglomerate, and are oriented along the nanofibers. Therefore, the obtained composite is classified as a 1-3-type nanocomposite with the nanowires aligned in a matrix [87]. The parallel arrangement of the fibers in the mats results from the use of the drum collector. Additional information on the production of PAN/SbSI, and its properties, can be found in [83].

### 2.3. Cellulose/SbSI Composite

**Figure 4:**
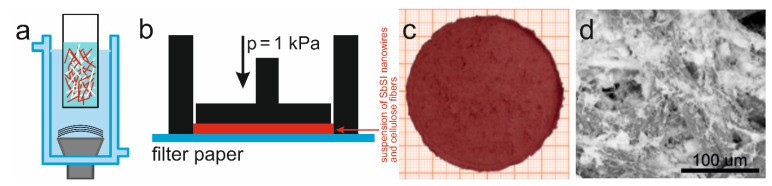
Schematic diagram for the fabrication of a cellulose/SbSI composite. (**a**) Ultrasonic mixing of the SbSI nanowires and cellulose fibers in water. (**b**) Drying of the homogeneous suspension that contains the SbSI nanowires and cellulose fibers, under a pressure of 1kPa. (**c**) Photograph and (**d**) SEM image of the obtained sheet of cellulose/SbSI nanocomposite.

The sonochemically produced SbSI nanowires and cellulose fibres (International Paper Co., Kwidzyn, Poland) were used to produce a cellulose/SbSI composite. The cellulose fibres, with transverse dimensions of 10–25 μm and a length of up to several millimetres, were sonicated (VCX-750 ultrasonics processor, 750 W, 565 W/cm^2^, 2 h) to obtain a homogeneous mixture in water. Then, the SbSI nanowires were added to obtain a weight ratio of 1:4 and the resultant mixture was sonicated once again for 2 h (Figure 4a). In the next step, the obtained suspension of SbSI nanowires and cellulose fibers was applied to a filter paper, then dried in a hand-made form under a pressure of 1 kPa (Figure 4b).

The product of the described process was a sheet of cellulose/SbSI nanocomposite, which had a thickness of about 0.05 mm (Figure 4c) and a SbSI nanowire content of 23 percent. This composite consists of the cellulose fibres with the SbSI nanowires uniformly dispersed among them (Figure 4d); it can be classified as a 0-3-type composite [87]. In this composite type, SbSI nanowires are randomly distributed within the cellulose matrix. For more detailed information on the composite morphology, and its fabrication, see [88].

### 2.4. Epoxy Resin/SbSI Composite

**Figure 5:**
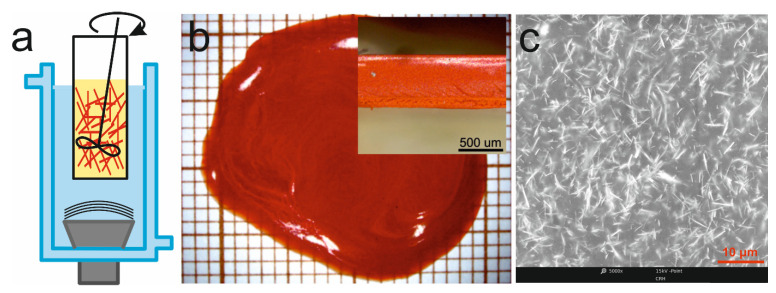
Schematic diagram for the fabrication of an epoxy resin/SbSI composite. (**a**) Preparation of a homogeneous mixture of SbSI nanowires, epoxy resin, and a hardener. (**b**) Photograph and (**c**) SEM image of the epoxy resin/SbSI composite. The inset of (**b**) displays the cross-section of the obtained composite plate.

SbSI nanowires were added to epoxy resin (LH288, HAVEL Composites, Praslavice, Czech Republic) in a 1:4 weight ratio (5 g SbSI nanowires, 20 g epoxy resin) to produce the epoxy resin/SbSI composite. To obtain a homogeneous mixture, the resultant composite was mixed intensively, mechanically first, and then ultrasound irradiation was used in the second stage (Figure 5a). The same processor and settings were used as previously, i.e., for the case relating to the production of a cellulose/SbSI composite. Once homogeneous, the hardener (H281, HAVEL Composites, Praslavice, Czech Republic) was added in a weight ratio of 1:4 with respect to the amount of the resin used. Then, the mixture was applied to a glass substrate and cured at a constant temperature (283 K) and relative humidity (5%) for 24 h. This created a flat plate of epoxy resin/SbSI composite with a thickness of 600 μm (Figure 5b). The SbSI nanowires were homogeneously distributed, albeit randomly, throughout the whole structure of the composite. Identical to the cellulose/SbSI composite, it is classified as 0-3-type composite [87]. Further information on its fabrication, properties, and morphology can be found in [89].

The synthesized epoxy resin/SbSI composite was used in the construction of a strain sensor. In the first construction, the epoxy resin/SbSI composite was used as a structural element of a FRP (Fibre Reinforced Polymer) laminate [90], while the second case was employed as a filling for a 3D printed skeleton grid [91] created by the FDP (Fused Deposition Modelling) method.

**Figure 6:**
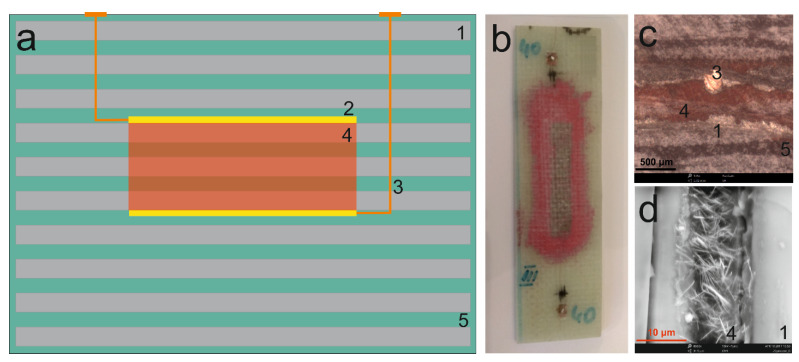
(**a**) Diagram depicting the scheme for the FRP laminate with built-in strain sensor and (**b**) a photograph of the synthesized laminate. (**c**,**d**) SEM images of the cross-section of the FRP laminate. Here, 1—plain-woven glass fabric, 2—silver electrodes, 3—copper wires, 4—epoxy resin/SbSI composite active layer, and 5—epoxy resin.

The FRP laminate consists of 10 layers (labelled 1 in Figure 6a) of a plain-woven glass fabric (305 g/m^2^, KROSGLASS, Poland). On the fourth and sixth layers were placed silver electrodes (2 in Figure 6a) with dimensions 10 × 60 mm, which were made by use of a high purity silver paste 05002-AB (SPI Supplies). Copper wires (3 in Figure 6a) were used as electrical leads that connect to contacts on the outer surface of the laminate. The epoxy resin/SbSI composite was applied to the surface of the silver electrodes, so that it covered an area that incorporated the fourth, fifth and sixth layers of the glass fabric (4 in Figure 6a). The two relevant layers were precisely joined to the electrode surfaces and the surface covered by the composite fitted perfectly into the three layers. The composite was pre-cured for an hour to form an active layer for sensing purposes. Then, using the VARTM (vacuum assisted resin transfer moulding) method, the 10 layers of glass fabric were saturated with a mixture of the same epoxy resin (LH288) and a hardener (H281). For more detailed information on the fabrication of the FRP laminate, with a built-in strain sensor, see [90].

**Figure 7:**
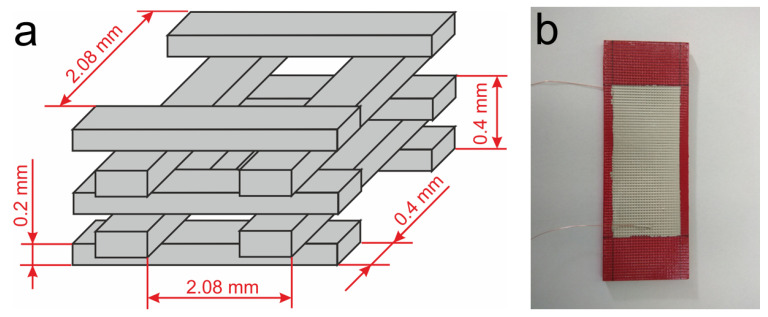
(**a**) Diagram showing the configuration of a single “cell” of a printed skeleton grid (with the dimensions provided) and (**b**) a photograph of the grid filled with epoxy resin/SbSI composite and the deposited silver electrodes.

The first stage in the construction of a sensor, based upon a 3D printed skeleton grid filled with epoxy resin/SbSI composite, was the printing of the grid by the FDM method (PRUSA MK3S printer, Prusa, Prague, Czech Republic) using polylactide (PLA, COLORFIL, Sosnowiec, Poland). The height of each grid component was 0.2 mm, while the corresponding thickness was 0.4 mm (see Figure 7a). In the next step, the prepared grid was filled with the epoxy resin/SbSI composite by gravity casting; the grid was completely covered with the composite. In the final step, silver electrodes with dimensions 40 × 100 mm were applied to the opposite sides of the skeleton. This involved using the same silver paste as previously used for the FRP laminate with built-in strain sensor. Copper wires were attached to the electrodes, also using the same paste, to connect the sensor to the measuring system. The detailed information on the fabrication of the described sensor can be found in [91].

### 2.5. PVDF/SbSI Composite

**Figure 8:**
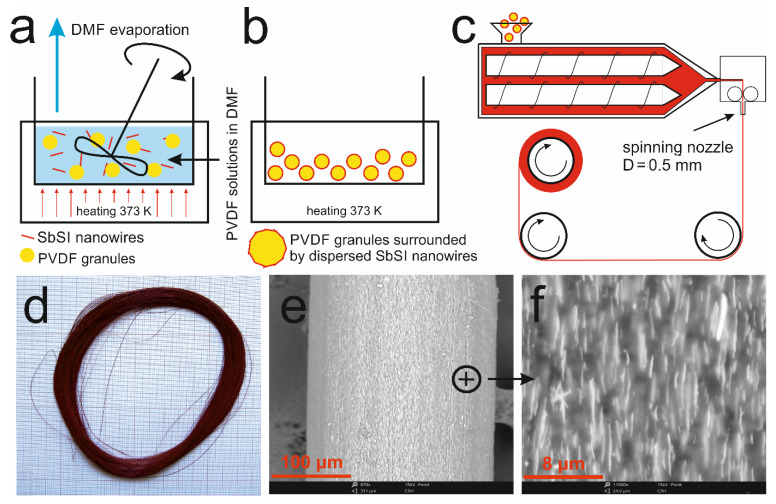
Images depicting the preparation scheme for PVDF/SbSI fibres. (**a**) Evaporation of DMF after the homogeneous suspension of the SbSI wires is obtained and an adding of 80 g of PVDF granules. (**b**) The obtained PVDF granules are covered by dispersed SbSI nanowires. (**c**) The twin-screw extruder that is used to form the fibres. (**d**) Photograph of the obtained PVDF/SbSI fiber for the case where the collection velocity is 200 m/s. (**e**,**f**) SEM images showing the fibre presented in Figure 8d.

During the preparation of the PVDF/SbSI composite, the following procedure was used to avoid agglomeration of the SbSI nanowires in the PVDF polymer. To begin, SbSI nanowires (15 g) were added to N,N-dimethylformamide (DMF, 20 g, Avantor Performance Materials Poland S.A., Gliwice, Poland), and the resulting mixture was intensively mixed in a closed container for 15 min, at a temperature of 373 K, using a magnetic stirrer. Next, polyvinylidene fluoride granules (PVDF, 5 g, Solvay Solexis SAS, Spinetta Marengo, Italy) were added to the vessel. Then, the mixture was stirred once again until the polymer was completely dissolved and a homogeneous suspension of SbSI wires was obtained. In the next step, a further quantity of PVDF (80 g) was added to the solution. The stirring continued until the surface of the PVDF granules was covered with the solution. After this stage, the vessel was opened and the stirring process was continued, at 373 K, until the DMF evaporated (Figure 8a). The obtained PVDF granules were covered by SbSI nanowires (Figure 8b). These were used to form the fibres using a twin-screw extruder (Zamak Mercator Sp. z o.o., Skawina, Poland). The melting zone of the extruder was at the temperature 453 K, while its extrusion zone was at 443 K. During the spinning process, a single-hole nozzle with a diameter of 0.5 mm was used and a pressure of 10 bar was applied (Figure 8c). The fibre stretch ratio was set at 20, while the rate of the fiber collection used were 0 m/s, 50 m/s, 100 m/s, 150 m/s, 200 m/s, 300 m/s, 500 m/s. As expected, the diameter of the produced fibres increased with a decrease in the collection velocity, e.g., 106 μm for 300 m/s, 162 μm for 150 m/s, and 224 μm for 50 m/s.

The PVDF/SbSI fiber obtained by use of a selected collection velocity (200 m/s) is presented in Figure 8d; its length is greater than 2000 m. Figure 8e,f are SEM images of the obtained fibre. We can see that the SbSI nanowires are scattered homogeneously within the PVDF matrix. Furthermore, the nanowires are arranged parallel to the long axis of the fibre, which is similar to the case of the PAN/SbSI composites. Thus, the obtained fibres are again classed as a 1-3-type nanocomposite [87]. The detailed information on the PVDF/SbSI composite, e.g., its optical and mechanical properties, and its WAXD (wide-angle X-ray diffraction) curves can be found in [92].

### 2.6. SbSeI Pellet

**Figure 9:**
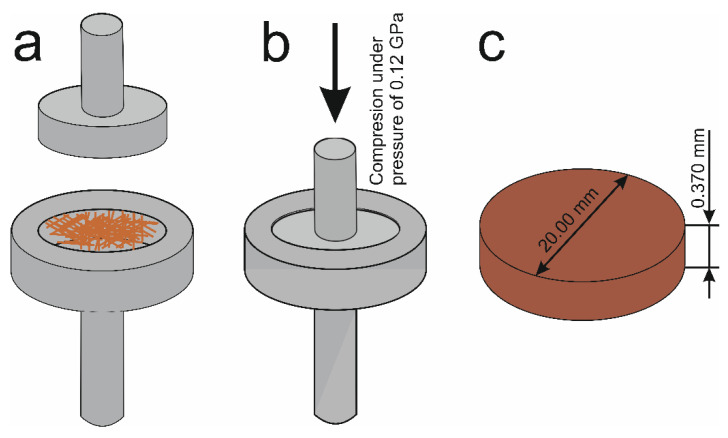
Schematic scheme for the fabrication of a SbSeI pellet: (**a**) filling the steel mould with the sonochemically produced SbSeI nanowires, (**b**) compression of the SbSeI nanowires under a high pressure, and (**c**) the dimensions of the created pellet.

The sonochemically prepared SbSeI nanowires were also used in the production of cylindrical pellets. SbSeI nanowires were placed in a steel cylinder (Figure 9a) and pressed together with a piston. In detail, the piston was pushed into the mold (Figure 9b) under a pressure of 0.12 GPa for 20 min [93] or 0.1 GPa for 120 s [94] using model 4469 of the testing machine (Instron, Norwood, USA). Both processes were performed at a temperature of 295 K. The obtained SbSeI pellet has a diameter of 20 mm and a thickness of 0.370 mm (Figure 9c). The application of a higher pressing pressure, over a longer period of time, resulted in a pellet with a greater mechanical strength and a greater compactness of the SbSeI nanowires (from 41% [94] to 50% [93]). More information about SbSeI pellet production, and its morphology, can be found in [93,94].

### 2.7. Measurements

**Figure 10:**
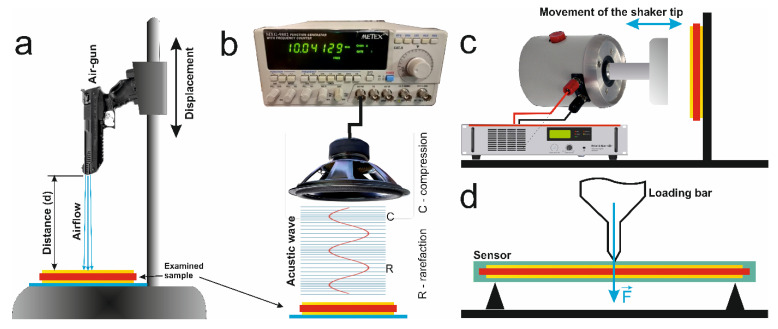
Diagrams showing various experimental set-ups that can be used for measuring the electrical response of the nanogenerators under the influence of (**a**) a shock wave, (**b**) an acoustic wave, and (**c**) an impact. (**d**) Scheme of a stand for measuring the piezoelectric response of the sensors for different applied loads.

The functional parameters of the nanogenerators were measured for different types of excitations: acoustic wave, shock pressure of air, impact, or strain. These measurements were attained using experimental set-ups dependent on the type of extortion (Figure 10). A dynamic signal analyser Photon+ (Bruel and Kjaer, Nærum, Denmark), a DS1202CA digital oscilloscope (RIGOL Technologies, Pekin, China), a Keithley 6517B voltmeter (Keithley, Solon, OH, USA), and an EG&G 5110 dual-phase lock-in amplifier (Princeton Applied Research, Oak Ridge, TN, USA) were used to record the electrical signals generated by the samples.

Figure 10a shows a setup used to measure the electrical response of the nanogenerators based on air shock pressure. The samples were mounted onto a tough surface, and an air gun Zoraki HP-01-2 (Atak Silah, Istanbul, Turkey) was positioned above them. The distance between the sample and the muzzle was changed by use of a vertical lever. The actual value of the shock wave pressure was measured with a WIKA S-10 pressure sensor (WIKA, Wloclawek, Poland) that was mounted at the position of the sample. The sensor measured the pressure in the range 0-100 bars with a precision of 0.1 V/bar.

The functional generator MXG-9802 (METEX, Seoul, Korea), and a loudspeaker were used to generate an acoustic wave (Figure 10b). The nanogenerators were mounted slightly under the loudspeaker on the testing table, thus the influence of any housing vibrations on the response was minimized. The acoustic pressure level was measured with a T-01 sound level meter (Sonopan, Białystok, Poland). An inductor attached to a plexiplate was used to generate the vibration in the 16–24 Hz range. The sample was mounted onto the plate with wax. A WH-30 vibrometer and an FO-1 octave filter (Stanmark Products, Cracow, Poland) were used to measure the vibrational frequency and the amplitude. The electrical responses of the nanogenerators due to an impact were measured using a Linear Power Amplifier (LPA100, Bruel and Kjaer) and a permanent magnetic shaker (LDS V201, Bruel and Kjaer). The test samples were placed onto a stiff surface, which is positioned perpendicular to the moving shaker tip (Figure 10c). The acceleration of the striking tip was also recorded with a Delta Tron Accelerometer (Type 4507 B 001; Bruel and Kjaer) that is mounted onto the tip. The sample and accelerometer signals were measured simultaneously. To register the piezoelectrical response of the sensors, under the influence of a strain, 3-point non-destructive bending tests (Figure 10d) were performed with an INSTRON 4469 testing machine (Instron, Norwood, MA, USA) that follows the PN-EN ISO 14125 standard.

## 3. Results

**Figure 11:**
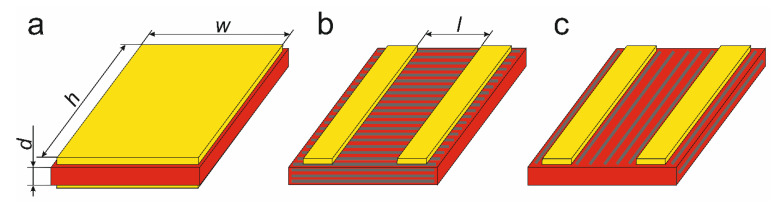
Drawings representing the two configurations of the examined samples: (**a**) sandwich-type and (**b**,**c**) planar-type. Here, *d* is the sample thickness, and *h*, *w* are the height and width of the sandwich-type samples surface, respectively, while *l* denotes the distance between the electrodes in the planar-type samples. In the case of the PAN/SbSI nanocomposites, the fibres can be arranged in either (**b**) a perpendicular or (**c**) a parallel direction compared to the electrodes in the planar-type samples.

The composite samples studied were prepared in two different configurations: either with the electrodes applied to opposite surfaces (a sandwich-type configuration, Figure 11a) or to the same surface (planar-type, Figure 11b,c). In the case of the PAN/SbSI composite mats (Figure 3c) and the planar-type samples, the fibres (and, therefore, the SbSI nanowires) were arranged perpendicular (Figure 11b) or parallel (Figure 11c) to the electrodes. The dimensions of all the studied samples are presented in Table 2.

### 3.1. Piezoelectric Nanogenerators

The greatest advantage of the piezoelectric nanogenerators (PENGs) is the direct conversion of mechanical energy into electricity. An energy potential in the piezoelectric materials appears when the central symmetry of the crystalline structure is broken under the influence of an external force. In its equilibrium state, the centres of the positive (cations) and negative (anions) charges in a crystal coincide with one another. During the deformation of the crystalline structure, on application of the external force, the charge centres of the cations and anions mutually shift from each other, which results in the formation of electric dipoles and, thus, a piezoelectric potential. When the deformed piezoelectric crystal is connected to an external circuit, a flow of electrons occurs that shields the piezopotential and, thus, the equilibrium state is re-established. The working principle of PENGs is based upon the change in the piezopotential due to the influence of the periodically changing deformation, which is accompanied by a flow of charges in the external electrical circuit.

**Figure 12:**
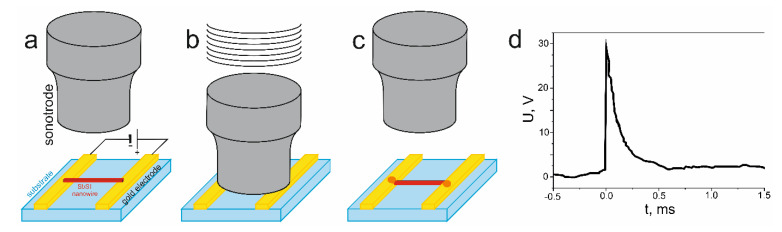
Scheme for the fabrication of a nanogenerator based on single SbSI nanowires: (**a**) alignment of the nanowires in an external electric field, (**b**,**c**) ultrasonic bonding of SbSI nanowires to the electrodes, and (**d**) the electrical response of the nanogenerator registered under a shock wave.

Mistewicz et al. described a construction method for a piezoelectric generator, which is based on single SbSI nanowires that operate under a shock wave [93]. In the first step, sonochemically prepared SbSI nanowires were dispersed within toluene in a ratio of 0.05 mg to 1 mL. Afterwards, a drop of the suspension was applied to a Si/SiO_2_ substrate with gold electrodes that are separated by 1 μm. The application occurred in the presence of an external electric field (*E* = 5 × 10^5^ V/m) that was generated between the electrodes (Figure 12a). Since the SbSI nanowires are ferroelectric, they were arranged perpendicular to the electrodes (see Figure 2 in [93] or Figure 2 in [94]). After it was dried, an ultrasonic bonding technique was used that improved the contact between the nanowires and the electrodes (Figure 12b,c). Due to the small size of the SbSI nanowires, a sonotrode with a SiC monocrystal at the end was employed. The smooth surface (with an average roughness of 2.6(3) nm) of the crystal enables a connection of the nanowires to the electrodes. The utilization of the bonding technique improved the mechanical strength of the structure and increased its conductivity by 420% [94]. The detailed information on fabrication using a sonotrode and the ultrasonic bonding technique, and the results of its application, can be found in [94].

The stand presented in Figure 10a, and a CP 88 air gun, were used to register the electrical response of the SbSI nanowires (sample S1 in Table 2) to the shock wave. The authors reported that the pressure from shock wave was 5.9 × 10^6^ Pa. However, this seems to be the pressure value for the CO_2_ cartridge and the actual measured pressure is 4.8 × 10^5^ Pa (4.8 bar). Using this value, and the diameter of the expanding gas stream (*D* = 4.5 mm), the force acting on the sample was calculated (*F* = 7.6 N). A curve of the electric field pulse generated from the fabricated nanogenerator under a shock wave, which is registered by a DS1202CA digital oscilloscope, is presented in [93]. With knowledge of the distance between the electrodes, these values were converted into the induced voltage during the gunshot (Figure 12d). The maximum value of the voltage was found to be 29 V.

The planar-type samples of the PAN/SbSI nanocomposites (samples S2 and S3) were prepared. These samples had similar dimensions to facilitate the comparison of the obtained results, and they are given in Table 2. The distinction between S2 and S3 was the different arrangement of the fibres and, thus, the SbSI nanocrystallites between the electrodes. In S2, the fibres are arranged perpendicular to the electrodes (Figure 10b), while in sample S3 they are parallel to them (Figure 11c).

**Figure 13:**
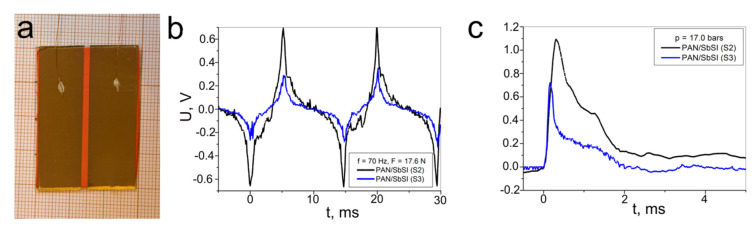
(**a**) Photograph of exemplary samples of the PAN/SbSI nanowires composites. (**b**) The output voltage from the PAN/SbSI samples over time, in which the black and blue lines relate to sample S2 and S3, respectively.These results are registered for (**b**) an impact with a force of 17.6 N at a frequency of 70 Hz and (**c**) a pressure of 17.0 bars.

Figure 13a is a photograph of the exemplary samples of PAN/SbSI (S2) with the fibres arranged perpendicular to the electrodes. The distance between the electrodes was identical for both samples (S2 and S3), because the same masking was used during the electrode sputtering, while there is a 1 mm length difference for the sample electrodes (see Table 2). The piezoelectrical response of the samples was recorded for two different types of excitations: an impact (Figure 10c) with a force of 17.6 N and a frequency of 70 Hz (Figure 13b) and a shock wave (Figure 10a) with a pressure of 17.0 bars (Figure 13c). The measured pressure can be converted into a force with knowledge of the expanding gas stream (*D* = 5.5 mm). Thus, the calculated force was found to be 40.4 N. Regardless of the type of excitation, the higher piezoelectric responses were recorded for the sample PAN/SbSI (S2) with fibres arranged perpendicular to the electrodes (Figure 13b,c) compared to the parallel alignment of the fibers with the electrodes (S3).

**Figure 14:**
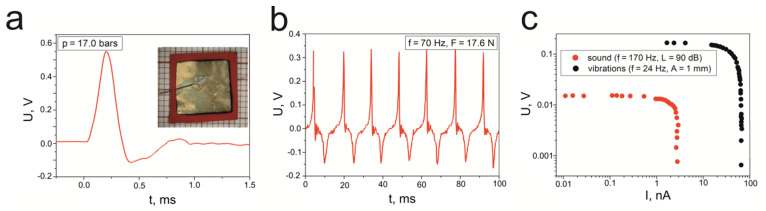
Graphs showing the output voltage from the epoxy resin/SbSI samples (S4), in which (**a**) the applied pressure is 17.0 bars and (**b**) the force impact is 17.6 N with a frequency of 70 Hz. (**c**) Graph depicting the output voltage of sample S4 against the current intensity, which are registered for different load resistances and various excitations. Here, the black spot represents an acoustic wave with a frequency of 70 Hz and a sound pressure level of 90 dB and the red spot denotes vibrations with an amplitude of 1 mm and a frequency of 24 Hz. The inset of (**a**) is a photograph of sample S4.

The inset of Figure 14a is a photograph of the epoxy resin/SbSI sample, which was created in the sandwich configuration (sample S4, initially described in [89]) with deposited gold electrodes. The sample surface was about 0.9 cm^2^, while its thickness was 500 μm. Figure 14a shows the electrical response of sample S4 under the influence of a shock wave with a pressure of 17.0 bars. The maximum recorded voltage was 0.55 V. The epoxy resin/SbSI sample was also checked for its response to impact. The obtained results for an impact force of 17.6 N, with a frequency of 70 Hz, are presented in Figure 14b. The graph shows several repeated cycles measured at a temperature of 293 K, and thus it displays very good signal repeatability with a peak-to-peak voltage (*U*_p–p_) of 0.5 V. Figure 14c provides the registered voltage signal from the epoxy resin/SbSI sample when acoustic waves (Figure 10b, red dot) and vibrations (black dots) are applied. In the case of acoustic waves, the presented results correspond to a sound frequency of 170 Hz and a sound pressure level of 90 dB, while for the vibrational excitation, the results were recorded at an applied frequency of 24 Hz and an amplitude of 1 mm. The obtained characteristics were also determined by use of various load resistances in a range from 10^4^ to 10^8^ Ω. The maximum voltages that were measured for the open circuit were 15 mV and 170 mV when the sound and vibrations were applied, respectively.

**Figure 15:**
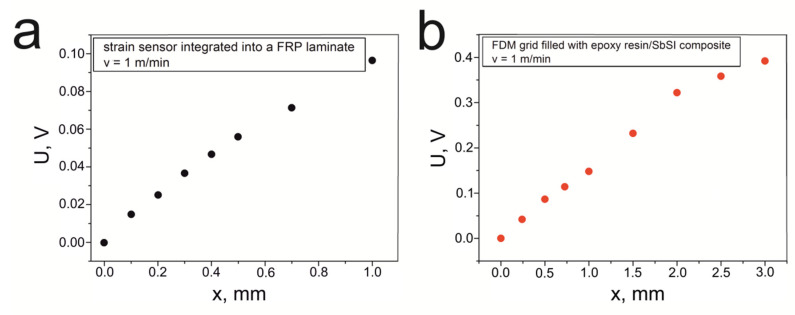
The maximum voltage for various deflections, and a constant deformation speed (*v* = 1 mm/min), for (**a**) the strain sensor integrated into an FRP laminate and (**b**) the FDM grid filled with the epoxy resin/SbSI composite.

Based on the epoxy resin/SbSI composite, two methods for constructing strain sensors were previously presented. The first structure was a sensor integrated into an FRP laminate (Figure 6, sample S5) [90], while the other one involved an FDM grid that was filled with the epoxy resin/SbSI composite (Figure 7, sample S6) [91]. To measure the piezoelectric response of the sensors, 3-point non-destructive bending tests were performed (Figure 10d). The distance between the supports was set at 200 mm and 140 mm for samples S5 and S6, respectively. The tests were performed for deflections that do not exceed the elastic strain range of a given sample. The maximum deflection for sample S5 was 1.0 mm, while S6 was 3 mm. Figure 15a,b shows the registered maximum voltage for various deflections, at a constant deformation speed (*v* = 1 mm/min), for the strain sensor integrated into an FRP laminate and the FDM grid filled with the epoxy resin/SbSI composite. For both sensors, the measured voltage is linearly dependent on the deflection value over the elastic deformation range that is studied.

**Figure 16:**
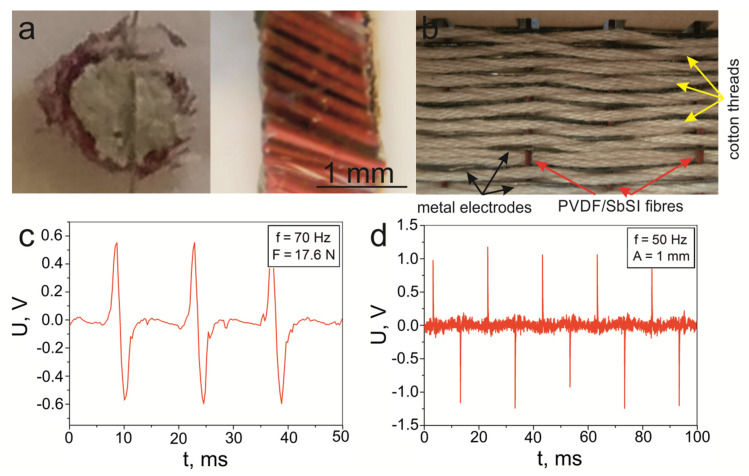
(**a**) Sample photograph consisting of around 300 PVDF/SbSI fibres that are arranged perpendicular to the electrodes (top and side views), (**b**) A textile fabric composed of interlacing fibres, metal wires, and cotton threads. (**c**) Voltage signal over time for the sample presented in Figure 16a under an impact (*f* = 70 Hz, *F* = 17.6 N, *T* = 293 K), (**d**) Voltage signal for the textile of (**b**) under a vibrational excitation (*f* = 50 Hz, *A* = 1 mm).

The fabrication (Figure 8) and the properties of PVDF/SbSI fibres were initially described in [92]. Figure 16a shows a sample composed of around 300 PVDF/SbSI fibers (collected with a velocity of 200 m/s), which are gathered and combined by use of silicon Elastosil N10 (DRAWIN Vertriebs GmbH, Hohenbrunn, Germany). The produced material was cut into plates so that sample S7 could be obtained, which contains a perpendicular orientation for the fibres compared to the electrodes (see the side view of Figure 16a). Fibres with a diameter of 80.7 μm were used. The cross-section of all the fibres in sample S7 is about 1.54 mm^2^, while its thickness is 1.18 mm (see Table 2). The fibres were collected with a lower speed (*v* = 50 m/min) and, therefore, a larger diameter (Φ = 204 μm) was used to fabricate the textile shown in Figure 16b. The textile fabric consists of a vertical weaving made from PVDF/SbSI fibres and a horizontal weaving composed of metal wires and cotton threads (sample S8). The metal wires operate as electrodes that collect the generated charges during the deformation of the fibres, while the cotton threads prevent short circuiting between the wires and enable the creation of a flexible textile.

The registered voltage signals from sample S7 due to impacts with a force *F*17.6 N and a frequency *f*70 Hz, at room temperature, is shown in Figure 16c. While the analogies results, in which a vibrational excitation of 50 Hz and an amplitude of 1 mm is applied, is given in Figure 16d. The determined values of the peak-to-peak voltage are 1.2 V under an impact, and over 2 V for applied vibrations. Very good signal repeatability can be observed for both samples and both excitation types.

**Figure 17:**
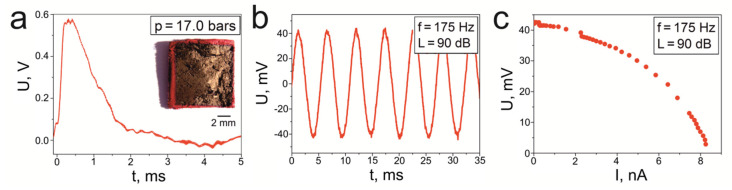
The output voltage from the cellulose/SbSI sandwich (sample S9) for (**a**) an applied pressure of 17.0 bars and (**b**) input acoustic waves with a frequency of 70 Hz and a sound pressure level of 90 dB (**c**) The output voltage from S9 against the current intensity for different load resistances and acoustic waves. The inset of (**a**) is a photograph of sample S9.

Figure 17 gives the output voltage from the cellulose/SbSI sample (S9) for two excitations: shock wave pressure (Figure 17a) and acoustic waves (Figure 17b). In the first case, the maximum voltage reached a value of 0.57 V for a pressure of 17.0 bars. The inset of Figure 17a shows a photograph of the cellulose/SbSI sample in a sandwich-type configuration with gold electrodes. The dimensions of sample S9 are provided in Table 2. In the case where the input of acoustic waves produces an electrical response from S9, the measured characteristic is presented in Figure 17b. The sinusoidal waves have a frequency of 175 Hz and a sound pressure level of 90 dB was used. It is clearly seen that the electric response of the sample has the same frequency as the input acoustic waves, while the peak-to-peak voltage in this case is 86 mV. Figure 17c shows the changing voltage, as a function of current intensity, under the acoustic wave excitations. The shape of the determined curve is characteristic of the source of electromotive force.

Toroń et al. described a SbSeI pellet (sample S10) (see Figure 9) as a generator of dynamic stimuli detection and a mechanical energy harvester [95]. The surface area and the thickness of an SbSeI pellet is 3.50 cm^2^ and 0.37 mm, respectively [95]. In this paper, the electrical response of S10 was measured under an impact and a finger pressure excitation. In the case of the impact excitation, an identical shaker (Figure 10c) was used. The maximum value of the peak-to-peak voltage reported in [95] is less than 400 mV under an impact excitation. The same parameter under a finger pressure is 3.41 V, while the average value for 10 cycles is 0.98 V (as reported in [95]). The shape of the determined curve is characteristic of the source of electromotive force. 

### 3.2. Pyroelectric Nanogenerators

In the case of the SbSeI pellet, not only is its piezoelectric properties useful [95] but also its pyroelectric properties. The latter has been investigated and a pyroelectric nanogenerator that recovers waste heat was constructed [96]. The electrical current is generated when there is a temperature gradient across opposite surfaces [96,97]. In the past, due to their low operation efficiency (around 2–5%), there was little interest in this type of device. However, recently, due to the continued development of nanotechnology, there are reports that devices can reach at least 15% efficiency (e.g., as presented in [98]) and, in future, similar devices could efficiently convert thermal energy into electricity.

In [95], the authors provide measurements of the voltage and current that are generated during heating and cooling cycles at one of the surfaces of the SbSeI pellet (sample S11). The cooling process was completed when the initial temperature was reached. Measurements were performed at near room temperatures, which implies low-grade waste heat (T < 373 K) with a slight temperature difference across the sample surfaces. It is noteworthy that more than 50% of total industrial waste heat resides in this range [99,100,101]. The maximum temperature difference was found to be 10 K. The most promising results were obtained when the initial sample temperature was set at 324 K and one of its surfaces was heated by 10 K, at a temperature rate of 0.2 K/s. The maximum value of the voltage and current were discovered to be 12 mV and 11 nA, respectively.

## 4. Discussion

The measurement of the electrical signal enables us to calculate the electrical surface power density, *P_S_*. In previously cited works, the power was calculated based on a variety of dependencies. In this study, an identical calculational method was used for all the obtained samples using the relationship [95,102]:

(1) $$P_{S}=\frac{1}{St_{R}R_{L}}\int_{0}^{t_{R}}U^{2}dt$$

where *S* denotes an active surface area, *R_L_* is a load resistance, and *t_R_* is a duration of the piezoelectric response. The active area is defined as the part of the sample area that is shared with the cross-sectional area that depends on the type of excitation. For example, for shock waves and impacts this is the area of the air stream and the surface of the end of shaker tip, respectively. All the values that are used in the calculations of the surface power density, and the obtained results, are shown in Table 3.

In the case of the PAN/SbSI composite (samples S2 and S3), the obtained results for impact and shock wave excitations, as well as for the different arrangement of nanofibers with respect to the electrodes, is presented for the first time (Figure 13b,c). We can see that optimal results, both in terms of the registered voltages (Figure 13b,c) and the calculated surface power density *P_S_* (Table 3), were obtained for nanofibers arranged perpendicular to the electrodes (sample S2) in comparison to PAN/SbSI composites (samples S3) with a parallel arrangement for the fibres.

The surface power density, *P_S_*, was also determined for the PVDF/SbSI fibres (sample S7) using Equation (1) (see Table 3) under an impact (*f* = 70 Hz, *F* = 17.6 N, *T* = 293 K). There is a significant difference between the value presented here (*P_S_* = 0.519 μW/cm^2^) and the one (*P_S_* = 408.8 μW/cm^2^) previously published [92]. There are two reasons for the difference. Firstly, the authors of [92] did not consider a filling of the impulses that are generated during the excitation. Secondly, and much more importantly, on converting the calculated power to a surface power density, they substituted the surface area of a single fibre instead of the whole sample of 300 fibres. The determined power density value, i.e., 0.519 μW/cm^2^ obtained for sample S7, is comparable to the value of 0.406 μW/cm^2^ for sample S2. The surface power density for the textile fabric, which is made by interlacing PVDF/SbSI fibres (sample S8), metal wires, and cotton threads (as seen in Figure 16b), is negligible. This is due to a marginal filling of the generated impulses, even despite a significant peak-to-peak voltage value (*U*_p–p_ = 2.3 V) with vibrational excitations (see Figure 16d).

The surface power density was also determined for other samples (epoxy resin/SbSI (S4), cellulose/SbSI sandwich (S9), and SbSeI pellet (S10)), in which the arrangement of the SbSI nanowires is random (see Table 2). In the case of samples S4 and S9, for applied sound waves and vibrations, the surface power densities were determined for different load resistances. Some were calculated for cases in which *R_L_* equals to 1 MΩ (which is the value used in all the other samples), while others relate to a load resistance for which the maximum signal was registered. These latter values correspond to the load resistance that matches the internal resistance of the samples. The respective *R_L_* values are presented in Table 3. Here, we notice a difference between the calculated value (*P_S_* = 0.057 μW/cm^2^) and the one (*P_S_* = 0.0141 μW/cm^2^) presented in [95] for a SbSeI pellet (sample S10). This is mainly due to an incorrect substitution in [95]; the sample resistance values were used instead of the load resistance, when substituting into Equation (1).

The most important conclusion, based on the calculations presented in Table 3, is that the arrangement of the nanowires in the produced composites is a crucial factor when creating efficient nanogenerators. As shown in [33,103], the piezoelectric coefficient *d*_33_ = 2000 pC/N is nearly 10 times larger than *d*_31_ and *d*_32_ for the SbSI single crystal. Moreover, the *d*_31_ and *d*_32_ coefficients have the opposite sign. All the presented samples were tested for two types of excitations: impact (except cellulose/SbSI sandwich (S9)) and shock wave (except SbSeI pellet (S10)). For both types of excitations, the highest values of the surface power density were obtained for PAN/SbSI and PVDF/SbSI composites, in which the fibres (and thus the SbSI nanowires) were arranged perpendicular to the electrodes. In future studies, the use of other types of excitations should also be considered, e.g., installation of an arc-shaped piezoelectric sheet between the outer race of rolling bearing and bearing pedestal [104] or using the household equipment vibrations (washing machine, food processor, etc.).

For piezoelectric strain sensors constructed with epoxy resin/SbSI nanowires composites (Figure 6 and Figure 7), their recorded electrical responses (Figure 15) are sufficiently large enough for potential application. These types of sensors could be used in SHM (structural health monitoring) systems to observe the condition of various types of advanced structures, e.g., load-bearing elements used in the yacht or aviation industry [105,106,107]. Their main task would be to ensure the safety of the structure through the continuous monitoring of the structural overloads. Currently, FRP laminates are commonly used in such constructions, although they can be damaged by delamination, and can be found in a wide range of applications. Usually, the sensor interferes with the structure and weakens it; however, this was not observed using the solution proposed in [90]. The registered deflection-test curves for the laminate samples with and without a sensor coincided. Following the destructive static bend tests, the values of the flexural strength and flexural modulus were obtained. A flexural strength and modulus value of 381(13) MPa and 19.7(6) GPa were found, respectively, for the sample with the sensor, while 393(19) MPa and 19.9(5) GPa were calculated without the sensor. We can see that the presented results are consistent with uncertainty. The range of applications for this type of sensor can be extended, since the FDM method can produce samples with virtually any shape.

A study on a SbSeI pyroelectric nanogenerator for low-temperature waste heat recovery has been described previously [96]. The maximum registered voltage and current values, and the conditions in which they were measured, were previously presented in Section 3.2. Based on these results, and using the sample size used by the authors, the surface power density for the presented device is *P_S_* = 0.059 × 10^−3^ μW/cm^2^. Unfortunately, this value is not competitive when compared to pyroelectric generators that are based on other materials; for example, the surface power density is much larger when PZT (lead zirconate titanate) is used, even by as much as four orders of magnitude [108,109,110,111,112]. Moreover, the determined value is an overestimation due to the applied methodology: the voltage measured for the open circuit and the short-circuit current were used in the calculations. However, it would be advisable to use SbSI nanowires in this type of construction. This suggestion is dictated by the difference in the pyroelectric coefficient for these materials, since this value is 44 × 10^−5^ C/m^2^K for SbSeI [96] and 12 × 10^−3^ C/m^2^K for SbSI [113]. As we can see, this parameter is much larger for SbSI. A major limitation of A^15^B^16^C^17^-type compounds in pyroelectric nanogenerators is the temperature range in which they can operate. These compounds are characterized by relatively low temperatures which they thermally decompose, e.g., above 623 K for BiSI [114] and above 545 K for SbSI [115].

In [88,89,92,95], the obtained power density values for A^15^B^16^C^17^ composites were compared with other devices. However, a fair comparison is very difficult for two reasons. First, there is no single standard measurement method for determining the parameters of nanogenerators. As a result, authors provide results for various types of excitations. For example, in [92] the results for the PVDF/SbSI nanowire composite obtained under an impact and vibration were compared with outcomes relating to bending [83,116,117], pressing [116,118], shock waves [93], and strain [119]. Even when authors provide results that use the same excitation type, they often measure different parameters; for example, in [83], the sample was bent at a frequency of 1 Hz, while [117] uses 0.2 Hz. In another case, [118] stated that the pressure force used was 2 N, while in [116] there is no comparable information about the force value. Secondly, there is no single calculational method for determining the power density. In this paper, Equation (1) is the key expression, while other studies (e.g., [118,120]) use the relationship *P_S_* = *U*^2^/*R_L_A* (here, *A* is an active surface area and *R_L_* is a load resistance). However, the latter relationship does not account for a filling of the generated peak, and it is based solely on the maximum value of the recorded voltage. In the near future, it seems necessary to develop a standard testing method for a nanogenerator, which would enable an accurate comparison of the obtained results.

The phenomenon called piezocatalysis can be observed in piezoelectric materials [121,122] and it is used to decompose various types of contaminations. In this type of application, the mechanical energy from ultrasonic waves is converted into a chemical energy or a piezopotential [123,124] or supports a photocatalysis [125,126]. When ultrasonic waves propagate a liquid, it is accompanied by a compression and a stretching of the chemical bonds within the medium, and thus a sound pressure of 10^5^ Pa [127] can appear. Simultaneously, during a period of negative pressure, the phenomenon of cavitation may result in the formation of cavitation bubbles. After reaching a critical size such bubbles collapse, which generates an enormous local pressure as high as 10^8^ Pa [128]. These phenomena can compress or bend SbSI nanowires and create a piezoelectric potential.

The use of piezoelectric nanomaterials is currently of particular interest; their abilities arise because they have a greater capacity for deformation compared to regular solid materials [127]. According to the piezoelectric effect, when an external stress is applied, piezocatalysis can occur during a deformation of the piezoelectric material. In such a scenario, an electric dipole moment is generated that has positive and negative charges at opposite sides of it. Since most piezoelectric materials have a wide energy gap (similar to insulators, broadband semiconductors), few free charge carriers arise when it is in its thermal equilibrium state (i.e., the charge carrier densities are 10^13^–10^20^ cm^−3^), so the induced dipole generates an independent electric field. As a result of this, two phenomena can occur: in one case, the energy level of the conductivity band (CB) is reduced below the highest occupied molecular orbital (HOMO) of the solution. This leads to an electron transfer from the HOMO to the CB. Alternatively, electrons transfer from the valence band (VB) to the lowest unoccupied molecular orbital (LUMO) of the solution [129,130]. If the concentration of charge carriers becomes higher, the piezopotential could be screened. Then, periodic excitation is required for a restoration of the electric field that enables the piezocatalysis [131]. The holes and electrons that are generated may participate in a redox reaction and produce reactive oxygen species (ROS); for example, the molecules ^●^$O_{2}^{-}$, ^●^OH, H_2_O_2_ may be formed [132,133]. The ROS that are created react with pollutants and degrade them.

The phenomenon of piezocatalysis, by use of a A^15^B^16^C^17^ compound is presented in [134]. In this work, SbSI nanowires are used to decompose the dye methyl orange (MO) in water. The tests were performed on an aqueous solution of MO in a concentration of 30 mg/L;the content of the SbSI nanowires was at the level of 6 g/L. Piezocatalysis was induced by an ultrasound device with different powers (750 W and 480 W) and frequencies (20 kHz and 40 kHz), respectively. In [134], the reaction kinetics rate constant, *k*, was set at 7.6 min^−1^ (for *f* = 20 kHz and *P* = 750 W) and 5.1 min^−1^ (for *f* = 40 kHz and *P* = 480 W). Such large values for the *k*-factor are due to the very rapid decomposition of MO in the presence of the SbSI nanowires under the influence of the ultrasound. In both cases, the time taken to decompose 99% of the MO did not exceed a minute. Comparing these values with results obtained from other nanomaterials, the performance of the SbSI nanowires is highly favorable; for example, the reaction kinetics rate constant can be double the ones for other piezocatalysts (see Table 4). Similarly high values for the reaction kinetics rate constants were found from other materials, but these decomposed another dye molecule Rhodamine B (RhB) [135,136,137]. Thus, a comparison between these cases is difficult, not only because different dye molecules were employed, but also due to the slightly lower values used for the ratio of the piezocatalyst mass to the mass of dye molecules that is given in these articles. Simultaneously, the results presented in [134] encourage additional research on the use of SbSI nanowires in the ultrasound-assisted degradation of different dye molecules. Testing the piezo-photocatalytic properties of the SbSI nanowires, which enable the degradation of pollutants, should also be considered in future work. Such tests are already popular for other materials; for example, Ag_2_O-BaTiO_3_ [138], Au-BaTiO_3_ [139], Al-BaTiO_3_ [140], CuS/ZnO [141], ZnO NWs [142], PZT-TiO_2_ [143], and ZnO nanorods [144]. This subject area is showing even more promise because other papers report very high values of the reaction kinetics rate constants for photocatalytic decomposition of MO using SbSI nanowires: 9 min^−1^ [134] and 25.2 min^−1^ [145].

## 5. Conclusions

An overview of the use of SbSI and SbSeI nanowires and their composites in various areas of application (i.e., piezo- and pyroelectric nanogenerators, piezoelectric sensors, and piezocatalysts) have been presented. In the case of nanogenerators, their crucial parameter (the power density *P_S_*) has been compared using the same calculational method for all the configurations introduced in this review. This method also allows the comparison of all the materials that are described in previous research, in which the power density was obtained from various mathematical relationships rather than one key equation.

For the first time, nanogenerators based on PAN/SbSI composites for perpendicular and parallel alignment of the SbSI nanowires relative to the electrodes have been presented. The influence of these arrangements in the produced composites on the value of the registered voltage and the power density has been examined. The best results have been obtained for nanogenerators in which the SbSI nanowires were arranged perpendicular to the electrodes (PAN/SbSI, PVDF/SbSI). A future consideration is whether technology can be developed that enable a reorganization of the A^15^B^16^C^17^ nanowires during the production of other composites (e.g., epoxy resin/SbSI). One potential method is to use their ferroelectric properties to orient them within an external electric field. It can be presumed that future devices of such types would have significantly improved the performance.

A nanogenerator based on SbSeI nanowires, and their pyroelectric properties has also been presented. It must be underlined that the power density obtained from them is currently unsatisfactory compared to other materials and, thus, requires further work. For example, it would be advisable to use SbSI nanowires (rather than SbSeI) in this type of nanogenerator due to a comparatively much higher value for its pyroelectric coefficient. However, an unavoidable limitation of the A^15^B^16^C^17^-type compounds related to the relatively low temperatures at which they thermally decompose and, therefore, the scope of their applicability may be restricted.

The sonochemically obtained SbSI nanowires have been extremely effective towards the decomposition of methyl orange under the influence of ultrasounds. The presented values for the reaction kinetics rate constant are two orders of magnitude larger than those for other piezocatalysts. However, similar values can be obtained for other catalysts that decompose rhodamine-B rather than methyl orange. Such studies must be continued and further evaluated, especially in connection to important industrial uses, e.g., the decomposition of pollutants in wastewater. Due to the strong photocatalytic properties of this material, it would be appropriate to study the degradation of contaminants under the influence of both ultrasound and light.

The FRP laminate with a built-in strain sensor, and the FDM grid filled with epoxy resin/SbSI composite, can be used to monitor construction conditions and prevent devastating structural failures. These issues are particularly relevant in bridge construction and the aviation and yacht industries. Since the FDM method is used in the printing of the grid, such sensors can be produced with practically any shape and, thus, could lead to a wide range of applications.

## Figures and Tables

**Table 1:** A comparison of the functional properties of a few selected piezoelectric materials, including SbSI single crystal and its ceramic equivalent.

Material	*T*_c_ [K]	*k*_33_	*d*_33_ [pC/N]
SbSI single crystal	288 [33]	0.8 [33]	2000 [33]
SbSI ceramic	292 [34]	0.72 [35]	650 [35]
Quartz	840 [36]	0.09 [36]	2.0 (d_11_) [36]
PZT-4	603 [37]	0.57 [37]	287 [37]
PZT-5	663 [37]	0.61 [37]	375 [37]
LiNbO_3_	1483 [37]	0.17 [37]	6 [37]
BaTiO_3_	393 [38]	0.52 [39]	73 [39]
95% BaTiO_3_ 5% CaTiO_3_	388 [36]	0.48 [36]	149 [36]
PbTiO_3_	743 [40]	0.28–0.30 [41]	140 [42]
PbZr_0.54_Ti_0.46_O_3_	646 [36]	0.62 [36]	152 [36]

**Table 2:** Dimensions and the electrical resistivity of the examined samples.

Sample Symbol	Sample Type	Orientation of Nanowires Relative to Electrodes	Thickness (*d*), mm	The Surface Area of the Sandwich-Type Samples (*h* × *w*), mm^2^	The Surface Area between the Electrodes (*l* × *h*), mm^2^	Ref.
S1	single SbSI nanowire on Si/SiO_2_ substrate		single nanowire (approximately 20–50 nm)	-	-	[93]
S2	PAN/SbSI	perpendicular	-	-	2.0 × 41.8	this paper
S3	PAN/SbSI	parallel	-	-	2.0 × 40.7	this paper
S4	epoxy resin/SbSI	random	0.5	90	-	[89]
S5	the strain sensor integrated into an FRP laminate	random	0.55	10 × 60	-	[90]
S6	FDM gride filled with epoxy resin/SbSI composite	random	3.0	40 × 100	-	[91]
S7	PVDF/SbSI fibers arranged perpendicular to the electrodes	perpendicular	1.18	1.54	-	[92]
S8	textile fabric	perpendicular	-	-	-	[92]
S9	cellulose/SbSI sandwich	random	0.05	8.6 × 9.1	-	[88]
S10	SbSeI pellet	random	0.37	25.67	-	[95]
S11	SbSeI pellet	random	0.37	188.6 ^a^		[96]

^a^—the area of the gold electrodes applied to the surfaces of the pellet.

**Table 3:** All the values used in the calculations of the surface power density and the obtained results. The symbols are defined in the text.

Sample Symbol	Sample Type	Orientation of Electrodes and Nanowires	*t_R_*, ms	*S*, cm^2^	*R_L_*, MΩ	*P_S_*, μW/cm^2^	Excitation	Ref.
S2	PAN/SbSI	perpendicular	14.2	0.100	1	0.406	impact	this paper
			3.1	0.053		3.974	shock wave	
S3	PAN/SbSI	parallel	14.2	0.100	1	0.111	impact	this paper
			3.1	0.053		0.736	shock wave	
S4	epoxy resin/SbSI	random	14.2	0.100	1	0.053	impact	this paper
			2.0	0.237		0.082	shock wave	
			5.9	0.9	2.90	0.022 × 10^−3^	acoustic wave	[89] *
			5.9	0.9	1	0.012 × 10^−3^	acoustic wave	[89] *
			41.7	0.9	2.50	0.042	vibrations	[89] *
			41.7	0.9	1	0.033	vibrations	[89] *
S7	PVDF/SbSI fibers	perpendicular	14.2	0.100	1	0.519	impact	[90] *
S9	cellulose/SbSI sandwich	random	3.1	0.237	1	0.226	shock wave	this paper
			5.9	1.00	1	0.011 × 10^−3^	acoustic wave	[88] *
			5.9	1.00	2.90	0.017 × 10^−3^	acoustic wave	[88] *
S10	SbSeI pellet	random	14.2	0.1	1	0.057	impact	[95]
			2.5	1.75		0.092	finger pressing	[95] *
S11	SbSeI pellet	random				0.059 × 10^−3^	heat	

*—value calculated using the Equation (1) based on the results presented in the cited articles.

**Table 4:** Comparison of the reaction kinetics rate constant, *k*, for different nanomaterials used for the ultrasound-assisted degradation of different dye molecules (methyl orange and rhodamine B) (abbreviations: *m*_p_/*m*_d_—the ratio of piezocatalyst mass to the mass of dye molecules, *P*/*P_S_*—power or power density of ultrasound, *f*—frequency).

Material	*k*, min^−1^	*P*/*P_S_*	*f*, kHz	Dye	*m*_p_/*m*_d_	Reference
SbSI NWs	7.6	750 W	20	MO	200	[134]
	5.1	480 W	40			
BaTiO_3_ NPs	19 × 10^−3^	80 W	40	MO	200	[146]
PLZT NWs	20 × 10^−3^	120 W	40	MO	200	[147]
Ba_1–x_Sr_x_TiO_3_ NWs	19.6 × 10^−3^	0.1 W/cm^2^	40	MO	200	[148]
MoSe_2_, NFs	3.45	250 W	40	RhB	20	[135]
MSe_2_, NFs	3.062	250 W	40	RhB	---	[136]
WS_2_, NFs	1.152	300 W	40	Rhb	50	[137]

NPs—nanoparticles, NFs—nanoflowers.

## Data Availability

Raw data were generated at Silesian University of Technology, Institute of Physics. The data presented in this study are available on request from the corresponding author.
